# Visual acuity after cataract surgery in Macular Telangiectasia Type 2 Stage 3 to 5

**DOI:** 10.1186/s40942-022-00386-0

**Published:** 2022-06-11

**Authors:** Jacob S. Heng, J. Fernando Arevalo, James T. Handa

**Affiliations:** 1 Department of Ophthalmology and Visual Science, Yale School of Medicine, New Haven, USA; 2 Department of Neuroscience, Johns Hopkins University School of Medicine, Baltimore, USA; 3 Wilmer Eye Institute, The Johns Hopkins Hospital, 400 N. Broadway, Smith 3015, Baltimore, USA

**Keywords:** Macular Telangiectasia, Cataract surgery, Ellipsoid zone

## Abstract

**Background:**

The purpose of this study was to evaluate visual acuity after cataract surgery in eyes with Macular Telangiectasia (MacTel) Type 2.

**Methods:**

Single-center retrospective cohort study of patients with MacTel Type 2 who underwent cataract surgery and were managed at the same institution. Patients underwent pre-operative assessment by a retinal specialist with examination and optical coherence tomography (OCT) at the same institution. The main outcome measure was the post-operative change in best corrected visual acuity (BCVA). Secondary study outcomes were achieving post-operative BCVA better than Snellen acuity of 20/40 and time to BCVA loss by two lines or more (10 or more ETDRS letters).

**Results:**

A total of 20 eyes (11 patients) underwent cataract surgery and were followed for a median of 25.5 months (IQR 17.5–44.2 months). The median post-operative BCVA improvement was 10.5 letters (IQR 3.50–20.25). Nuclear sclerosis severity [β = 8.99 (95% CI 3.35, 14.6), p = 0.00177] was associated with post-operative change in BCVA and central foveal ellipsoid zone (EZ) breaks [OR 1.33 × 10^–9^ (95% CI 5.12 × 10^–10^–3.43 × 10^−9^), p < 0.001] on OCT was inversely correlated with post-operative BCVA > 20/40 using a multivariate generalized linear model. Central foveal EZ breaks [HR 1.77 × 10^9^ (95% CI 3.86 × 10^8^, 8.11 × 10^9^), p < 0.001] and MacTel Type 2 disease stage [HR 2.83, (95% CI 1.12, 7.12), p = 0.027] were independently associated with shorter time to vision loss of two lines or more in a multivariate Cox regression model.

**Conclusions:**

Visual acuity significant improved after cataract surgery in eyes with MacTel Type 2 regardless of disease severity. The presence of central foveal EZ breaks may predict poorer post-operative visual acuity and subsequent vision loss from disease progression.

## Background

Macular telangiectasia (MacTel) Type 2 is a bilateral, progressive, neurodegenerative disease of uncertain etiology with characteristic vascular changes and apparent cell loss within the retina. The vascular changes are likely secondary to loss of retinal cells such as Muller glia [1–3]. Characteristic changes on optical coherence tomography (OCT) include hyporeflective spaces (“cavities” or cavitations) in the inner and outer retina, central retinal thinning and apparent loss of the photoreceptor ellipsoid zone [EZ, formerly known as the inner-segment (IS)/outer-segment (OS) junction line] [4] that appear as focal disruption of the EZ. Such disruption of the EZ likely represents loss of photoreceptor cells [3]. Generally, focal EZ disruption begins in the extra-foveal temporal macula, progressing to involve the foveal center and then the nasal macula. Progressive EZ loss has been associated with progressive focal loss of macular sensitivity measured by microperimetry [5, 6] and focal electrophysiological dysfunction measured by multifocal electroretinography [7]. EZ disruption in the foveal center is closely correlated with loss of best corrected visual acuity (BCVA) [6, 8, 9].

MacTel Type 2 typically presents after the fifth or sixth decade of life [4, 10], when the occurrence of visually-significant cataracts is also common [11]. In eyes with MacTel Type 2, like any other pre-existing macular pathology, the decision to proceed with cataract surgery is often a challenging one. Firstly, the benefit of cataract surgery in eyes with MacTel Type 2 is uncertain, given that any reduced visual acuity can be due to disruption of macular architecture by the MacTel Type 2 disease process rather than the cataract itself. Secondly, cataract surgery itself can potentially exacerbate pre-existing retinal disease. In a Phase II clinical trial of ranibizumab for non-neovascular Macular Telangiectasia Type 2 patients, one participant demonstrated marked increase in vascular leakage on fluorescein angiography and retinal thickness on OCT following cataract surgery [12]. However, the effect of cataract surgery on the progression of MacTel Type 2 has not been systematically studied. Therefore, the aim of this study was to evaluate visual acuity outcomes in patients with Macular Telangiectasia Type 2 following cataract surgery.

## Methods

### Patient selection

This study is a single-center retrospective cohort design of patients with Macular Telangiectasia Type 2 (MacTel Type 2) who had undergone cataract surgery and were managed at the Wilmer Eye Institute between July 1, 2013 and June 30, 2018. Approval by the local institutional review board was obtained for all study-related acquisition and HIPAA regulations were followed. Inclusion criteria included (i) a clinical diagnosis of Macular Telangiectasia Type 2, based on findings of fundoscopy, OCT and fluorescein angiography; (ii) an eye examination and a Macula OCT performed at the same institution before cataract surgery; and (iii) at least 3 weeks of follow up with a final post-operative manifest refraction.

Exclusion criteria included concurrent vitreoretinal surgery at the time of cataract surgery, any corneal pathology or other media opacity that affected the visual axis, and initiation of intravitreal VEGF-inhibitors within 6 months of cataract surgery.

### Study parameters

The patients’ characteristics including age at surgery, sex, ethnicity, and comorbid medical conditions were recorded. The best corrected visual acuity (BCVA) was measured by transilluminated distance Snellen or Early Treatment Diabetic Retinopathy Study (ETDRS) charts. Recorded Snellen BCVA was converted to approximate ETDRS letters [13] for analysis and reporting.

Disease staging of macular telangiectasia was carried out using the classification system proposed by Gass and Blodi [14] following evaluation by a retina specialist. Cross-sectional OCT images were examined for ellipsoid zone (EZ) break(s) in the foveal center. An EZ break is defined as a disruption in the continuity of the hyper-reflective layer representing the EZ on OCT. The foveal center on OCT is defined by deepest central point in the foveal pit marked by the absence or near absence of the inner nuclear layer (INL) and the relative enlargement of the outer nuclear layer (ONL) [15]. Early macular OCT images were taken using Time Domain (TD)-OCT (Stratus; Zeiss, Jena, Germany). Subsequent macular OCT images were taken using Spectral-Domain (SD)-OCT either on the the Spectralis HRA-OCT (Heidelberg Engineering, Germany) or the Cirrus HD-OCT (Carl Zeiss Meditec, Jena, Germany). We therefore decided to use a categorical grading system rather than quantifying the EZ loss due to the potential variability in EZ detection between these systems.

Cataract grading was carried out with slit lamp examination by the operating cataract surgeon according to the WHO grading system [16].

### Study outcome measures

The primary study outcome was the change in best corrected visual acuity (BCVA) after cataract surgery. The BCVA recorded at the pre-operative examination and the post-operative BCVA after manifest refraction recorded between 3 weeks and 2 months following cataract surgery were used for analysis.

Secondary study outcomes were achieving post-operative BCVA better than Snellen acuity of 20/40 and time to BCVA loss of 2 lines or more (10 or more ETDRS letters). In determining BCVA loss in patients who developed posterior capsular opacification, only the final visual acuity after YAG capsulotomy was taken into account.

### Statistical analysis

Statistical analysis was carried out using R version 3.5.2. A p-value of less than 0.05 was considered statistically significant. The same variables were analyzed for the primary and secondary study outcomes. Because most patients had both eyes included, statistical procedures utilizing generalized estimating equations (GEE) were used to account for the correlation between both eyes from the same patient. In GEE, dependence within clusters (eyes) are accounted for by estimating population-averaged effects. For continuous and categorical dependent variables, GEE-incorporated generalized linear and logistic regression were carried out using the “PGEE” package [17]. Variables found to be statistically significant on univariate analysis were incorporated into the corresponding multivariate models. For survival analysis, Cox regression with the “cluster” function was carried out for each variable using the “survival” package [18]. The “cluster” function is analogous to GEE in accounting for correlation between both eyes from the same patient. Statistically significant variables on univariate clustered Cox regression were then entered into a multivariate clustered Cox regression model.

## Results

A total of 20 eyes of 11 patients underwent phacoemulsification cataract surgery using the Alcon Infiniti Vision System (Alcon, Geneva, Switzerland) during the study period. All eyes were implanted with acrylic monofocal intraocular lenses. No toric or multifocal lenses were used. Sex and ethnicity were excluded from subsequent multivariate analyses due to the small number of male (1 male, 10 female) and Asian patients (1 Asian, 10 White or Caucasian), respectively. Median follow-up was 25.5 months (IQR 17.5–44.2 months) and mean follow-up was 40.0 months. The median age at the time of cataract surgery was 69.0 years (IQR 63.7–78.0 years). Three eyes had stage 3 disease, 15 eyes had stage 4 disease, and 2 eyes had stage 5 disease. Thirteen out of 20 eyes (65%) had evidence of central foveal EZ breaks at baseline.

**Fig. 1:**
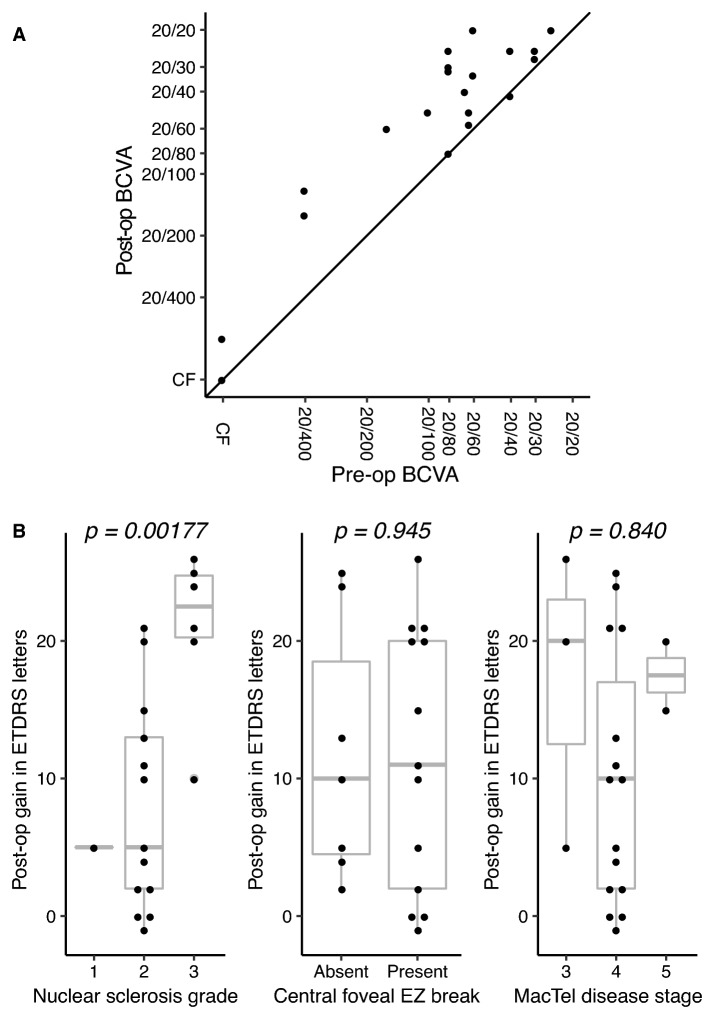
**A** Scatterplot showing best corrected visual acuity (BCVA) before and after cataract surgery. Pre-operative Snellen BCVA is plotted on the x-axis and post-operative Snellen BCVA is plotted on the y-axis. The diagonal line intersecting the origin represents no change in BCVA. Values above the diagonal line represent an increase in visual acuity and vice versa. **B** Dot and box plots showing post-op gain in ETDRS letters according to (left) nuclear sclerosis grade, (center) absence or presence of central foveal ellipsoid zone (EZ) break, and (right) MacTel disease stage

The median improvement in ETDRS letters post cataract surgery was 10.5 letters (IQR 3.50–20.25, p = 0.0003) (Fig. 1A). In univariate generalized linear models, age [β = −0.61 (95% CI −1.01, −0.21) p = 0.00275], sex [β = 14.1 (95% CI 10.0, 18.1), p < 0.001], ethnicity [β = −5.94 (95% CI −10.3, −1.63), p = 0.007] and nuclear sclerosis (NS) grade [β = 10.9 (95% CI 6.62, 15.2), p < 0.001] were significantly associated with post-operative change in BCVA (Table 1, Fig. 1B). Presence of central foveal EZ breaks and MacTel disease stage were not significantly associated with post-operative change in BCVA (Fig. 1B). When age and NS grade were entered into a multivariate generalized linear model, only NS grade [β = 8.99 (95% CI 3.35, 14.6), p = 0.00177] was significantly associated with post-operative change in BCVA.

**Table 1:** Gain in ETDRS letters according to patient and ocular characteristics

Characteristic	No. of eyes	Gain in ETDRS letters after cataract surgery	Interquartile range	p-value
Patient characteristics				
Age (years)				
≤ 69 years	11	15.0	10.0–20.5	0.00275**
> 69 years	9	4.00	2.00–13.00	
Sex				
Female	19	10.0	2.0–20.0	< 0.001**
Male	1	25.0	–	
Ethnicity				
White or Caucasian	18	10.5	2.5–20.0	0.007**
Asian	2	17.0	–	
Hypertension				
Absent	4	12.5	8.75–16.25	0.804
Present	16	10.5	2.0–21.0	
Diabetes mellitus				
Absent	11	5.0	1.0–20.5	0.416
Present	9	13.0	10.0–20.0	
Ocular characteristics				
Pre-op BCVA (ETDRS letters)				
> 57 ETDRS letters	10	5.0	2.5–10.8	0.361
≤ 57 ETDRS letters	10	20.0	11.2–21.0	
MacTel Disease Stage				
Stage 3	3	20.0	12.5–23.0	0.840
Stage 4	15	10.0	2.0–17.0	
Stage 5	2	17.5	–	
OCT findings				
Central foveal EZ break				
Absent	7	10.0	4.5–18.5	0.945
Present	13	11.0	2.0–20.0	
Pre-op central subfield thickness (μm)				
≤ 216 μm	10	15.0	2.5–20.8	0.950
> 216 μm	10	10.5	5.0–14.5	
Cataract grading				
Nuclear sclerosis				
Grade 1	1	5.0	–	0.00177**
Grade 2	13	5.0	2.0–13.0	
Grade 3	6	22.5	20.2–24.8	
Cortical cataract				
Grade 0	10	17.5	6.25–20.75	0.057
Grade 1	2	13.0	–	
Grade 2	3	10.0	5.0–17.0	
Grade 3	5	2.0	2.0–11.0	
PSC				
Grade 0	15	13.0	4.5–20.5	0.500
Grade 1	3	0.0	−1.0–5.0	
Grade 2	2	13	–	

P value of univariate generalized linear model with generalized estimating equation for each variable is shown

Age, pre-op BCVA and pre-op central subfield thickness were analyzed as continuous variables but categorized into above and below median values for display in this table

*p-value of < 0.05

**p-value of < 0.01

A total of nine out of 20 eyes (45%) achieved post-operative BCVA of better than 20/40 (Table 2). On univariate logistic regression, pre-operative BCVA [OR 1.12 (95% CI 1.02–1.23), p = 0.0131] and presence of central foveal EZ breaks [OR 5.62 × 10^–10^ (95% CI 2.89 × 10^–10^–1.09 × 10^−9^), p < 0.001] were positively and negatively associated with achieving post-operative BCVA of better than 20/40, respectively. When entered into a multivariate logistic regression model, only the presence of central foveal EZ breaks [OR 1.33 × 10^–9^ (95% CI 5.12 × 10^–10^–3.43 × 10^−9^), p < 0.001] was statistically significant, and showed an inverse correlation with achieving post-operative BCVA of better than 20/40.

**Table 2:** Patient and ocular characteristics of eyes achieving post-op BCVA of better than 20/40 or 20/40 or worse

Characteristic	All	Post-op BCVA > 20/40	Post-op BCVA ≤ 20/40	Odds ratio [95% CI]	p-value
Patient characteristics					
Age (years) [IQR]	69.0 [63.8–78.0]	69.0 [62.0–72.0]	69.0 [65.0–78.0]	0.96 [0.83, 1.11]	0.568
Sex (eyes)					
Female	19	8	11	–	1.00
Male	1	1	0		
Ethnicity (eyes)					
White or Caucasian	18	8	10	0.80 [0.29, 2.23]	0.669
Asian	2	1	1		
Hypertension (eyes)					
Absent	4	3	1	0.20 [0.03, 1.67]	0.136
Present	16	6	10		
Diabetes mellitus (eyes)					
Absent	11	4	7	2.19 [0.38, 12.62]	0.381
Present	9	5	4		
Ocular characteristics					
Pre-op BCVA [IQR] (ETDRS letters)	57.0 [47.5–63.2]	61.0 [55.0–76.0]	50.0 [20.0–59.5]	1.12 [1.02, 1.23]	0.0131*
MacTel Disease Stage (eyes)					
Stage 3	3	1	2	1.45 [0.40, 5.29]	0.573
Stage 4	15	7	8		
Stage 5	2	1	1		
OCT findings					
Central foveal EZ break (eyes)					
Absent	7	7	0	5.62 × 10^–10^ [2.89 × 10^–10^, 1.09 × 10^–9^]	< 0.001**
Present	13	2	11		
Pre-op central subfield thickness [IQR] (μm)	216 [190–256]	212 [167–233]	221 [194–278]	0.99 [0.97, 1.01]	0.308
Cataract grading					
Nuclear sclerosis (eyes)					
Grade 1	1	0	1	1.71 [0.51, 5.68]	0.383
Grade 2	13	6	7		
Grade 3	6	3	3		
Cortical cataract (eyes)					
Grade 0	10	6	4	0.74 [0.32, 1.74]	0.491
Grade 1	2	0	2		
Grade 2	3	1	2		
Grade 3	5	2	3		
PSC (eyes)					
Grade 0	15	7	8	0.93 [0.19, 4.55]	0.930
Grade 1	3	1	2		
Grade 2	2	1	1		

Odds ratio, 95% confidence interval (CI) and p-value of logistic regression with generalized estimating equations for each variable are shown

* denotes a p-value of < 0.05

** denotes a p-value of < 0.01

**Fig. 2:**
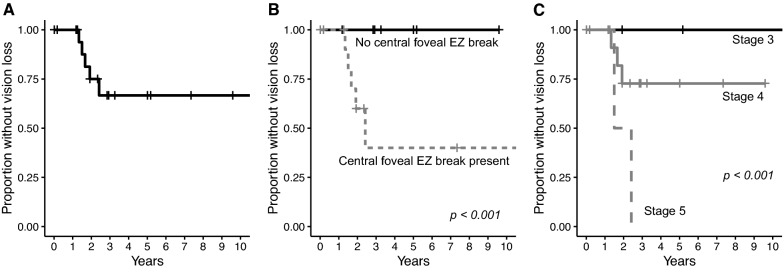
Kaplan–Meier curves of vision loss of 2 lines or more (10 or more ETDRS letters) in eyes with MacTel Type 2 after cataract surgery. **A** Kaplan–Meier curve of all eyes. **B** Kaplan–Meier curves of eyes with no central foveal ellipsoid zone (EZ) break (solid black line) and eyes with central foveal EZ break (dashed grey line). **C** Kaplan–Meier curves of eyes with MacTel Stage 3 (solid black line), Stage 4 (solid grey line) and Stage 5 (dashed grey line). p-values for univariate Cox regression are shown

No intra-operative complications were encountered. A total of 6 out of 20 eyes (30%) developed visually-significant posterior capsular opacification that required YAG capsulotomy. Other post-operative complications including pseudophakic cystoid macular edema, did not develop in the post-operative period. Five out of 20 patients had significant vision loss, defined by a decrease in visual acuity of two lines or more (10 or more ETDRS letters), during the follow-up period starting at 16 months post-surgery. On survival analysis, 66.7% of patients did not have significant vision loss (Fig. 2A). The median decrease in visual acuity per year was 0.40 letters (IQR 0–3.05), with a mean of 2.31 letters. On univariate Cox regression, sex [HR 1.33 × 10^–8^ (95% CI 1.47 × 10^–9^, 1.2 × 10^–7^), p < 0.001], disease stage [HR 5.36, (95% CI 2.36, 12.1), p < 0.001] and presence of central foveal EZ breaks [HR 1.67 × 10^9^ (95% CI 3.56 × 10^8^, 7.81 × 10^9^), p < 0.001] were significantly associated with vision loss over time (Fig. 2B, C). When disease stage and the presence of central foveal EZ breaks were entered into a multivariate Cox regression model, both disease stage [HR 2.83, (95% CI 1.12, 7.12), p = 0.027] and presence of central foveal EZ breaks [HR 1.77 × 10^9^ (95% CI 3.86 × 10^8^, 8.11 × 10^9^), p < 0.001] were significantly associated with a shorter time to vision loss. Qualitatively, all eyes with significant vision loss had progression of EZ disruption on OCT, although EZ loss could not be reliably quantified due to the different OCT platforms used in different patients.

## Discussion

In this study, we examined the visual acuity of patients with Macular Telangiectasia (MacTel) Type 2 following cataract surgery. Firstly, we showed that the majority of eyes with MacTel Type 2 had a significant gain in visual acuity (median of 10.5 ETDRS letters) in the early post period following cataract surgery. While not unexpected, the magnitude of improved visual acuity in these eyes correlated with the clinical severity of the nuclear sclerotic cataract. To the best of our knowledge, no study has examined the improvement in visual acuity following cataract surgery in MacTel Type 2 patients.

Secondly, we found that the presence of central foveal ellipsoid zone (EZ) disruption on OCT was significantly associated with MacTel Type 2 eyes not achieving a post-operative visual acuity of better than 20/40 in the 2 months following cataract surgery. While the initial clinical staging of MacTel Type 2 by Gass and Blodi [14] focused on the vascular changes, it is now recognized that these vascular changes result from loss of retinal cells such as Muller glia [1–3], which manifests as retinal cavitations on OCT. Experimental evidence indicates that oxidative stress, combined with impaired antioxidant pathways (e.g. serine synthesis), induces retinal cell death [19]. Indeed, we have previously shown that mice with hypoxia-induced oxidative stress coupled with impaired serine synthesis had increased cell death and developed retinal cavitations reminiscent of MacTel Type 2 [20]. With MacTel Type 2, retinal cavitation volume correlates with disease progression and precedes EZ loss [21], which presumably correlates with photoreceptor damage or loss [3]. Peto et al. [8] showed that about 76% of MacTel Type 2 patients without initial EZ disruption developed focal EZ loss after 5 years of follow up. To date, EZ loss is the most established surrogate biomarker for visual function in MacTel Type 2 because EZ loss has been associated with loss of retinal sensitivity as measured by microperimetry [5, 6], which precedes a loss in visual acuity [6, 8]. When EZ loss affects the foveal center, visual acuity [8, 9]. This observation is consistent with our finding of post-operative visual acuity of 20/40 or lower being associated with central foveal EZ breaks. These results suggest that assessing central foveal EZ disruption in eyes with MacTel Type 2 prior to undergoing cataract surgery is a valuable prognostic indicator of post-operative visual potential.

Finally, we found that the improved visual acuity following cataract surgery was maintained in the majority (66.7%) of MacTel Type 2 eyes during a median follow-up period of 25.5 months and a mean follow-up of 40.0 months. However, MacTel Type 2 eyes with central foveal EZ disruption on pre-operative OCT were more likely to experience loss in visual acuity of two lines or more (the equivalent of 10 or more ETDRS letters) after the initial improvement following cataract surgery. Conceivably, vision loss in MacTel Type 2 eyes could result from complications arising after cataract surgery. In a Phase II clinical trial of ranibizumab for non-neovascular Macular Telangiectasia Type 2 patients, one participant demonstrated marked increase in vascular leakage on fluorescein angiography and retinal thickness on OCT within 4 weeks of undergoing cataract surgery, which was attributed to surgery-induced pseudophakic cystoid macular edema (Irvine-Gass syndrome) [12]. In our study, we did not observe pseudophakic cystoid macular edema or any other retinal complications from cataract surgery. Rather, vision loss was likely to have resulted from disease progression. In our cohort, 5 out of 20 eyes had significant vision loss of 2 lines or more. The annual loss in visual acuity was positively skewed by a few patients, with a median of 0.40 letters and a mean of 2.31. These figures are slightly higher when compared to the vision loss described in other studies examining the progression of visual acuity loss in MacTel Type 2. The slightly higher rate of vision loss observed in our study could be due to the higher percentage of patients with central foveal EZ breaks at baseline (65%) compared to previous studies. Sallo et al. [5] found a mean annual loss of 0.267 letters, with 12 out of 39 eyes (30.8%) having central foveal EZ breaks. Heeren at el. found a mean annual vision loss of 2.2 letters, although the percentage of patients with central foveal EZ breaks was not reported [22]. Peto et al. [8] found 27% of eyes to have lost two lines or more at 5 years of follow-up and a yearly loss of 1.07 letters, with 372 out of 974 eyes (38%) having central foveal EZ breaks at baseline. We found that central foveal EZ breaks at baseline was significantly associated with a shorter time to vision loss of two lines or more, which is likely due to the progression of the underlying disease process affecting the fovea. Therefore, the higher prevalence of central foveal EZ breaks in our cohort at baseline, rather than cataract surgery, is more like to account for the apparently higher rate of vision loss.

Interestingly, we found that disease severity, as proposed by Gass and Blodi [14], was associated with a shorter time to vision loss of two lines or more after cataract surgery in eyes with in MacTel Type 2 independent of the presence of central foveal EZ breaks. In a previous study by Heeren et al. [6], advanced stages of MacTel Type 2 were associated with the presence and size of an absolute scotoma on microperimetry; the presence of a scotoma was in turn associated with progressive functional decline on microperimetry. The presence of intraretinal clumping on examination, which is a feature of stage 4 disease in the Gass and Blodi staging system [14], is likely to be a less sensitive marker for photoreceptor damage than EZ breaks on OCT and therefore, an indication of more severe photoreceptor loss.

The limitations of our study include its retrospective design and small sample size. However, due to the relative rarity of MacTel Type 2, a prospective study for our study aim would not have been feasible. Another limitation is that we could only qualitatively, and not quantitatively, analyze central foveal EZ breaks due to the different OCT platforms used for collecting images in different patients. Finally, the grading of cataracts was not carried out by standardized photographs but by clinical slit-lamp examination, which could be prone to inter-observer variability. A relative strength of our study is that the patients had their pre-operative evaluation, cataract surgery and post-operative follow up completed at the same institution, which eliminates variations in clinical practice that could exist between different institutions.

## Conclusions

We found that the majority of MacTel Type 2 eyes had significant gains in visual acuity following cataract surgery regardless of the underlying MacTel disease severity, as measured by the Gass and Blodi staging system [14] or the presence of central foveal EZ breaks. The gain in visual acuity was correlated with the severity of the cataract determined by clinical slit-lamp examination and was maintained in the majority of patients. However, the presence of central foveal EZ breaks on OCT was correlated with post-operative vision of 20/40 or worse and a shorter time to vision loss following cataract surgery that is likely to be a consequence of disease progression.

## Data Availability

The datasets generated and/or analysed during the current study are not publicly available due to institutional regulations in accordance with the Health Insurance Portability and Accountability Act (HIPAA) Privacy Rule, but are available from the corresponding author on reasonable request.

## Abbreviations

BCVA: Best corrected visual acuity
IQR: Interquartile range
OR: Odds ratio
HR: Hazard ratio
CI: Confidence interval
